# Perception of inherited risk in type 2 diabetes: a systematic review

**DOI:** 10.3389/fpubh.2023.1293874

**Published:** 2023-12-14

**Authors:** Elisa Airikkala, Mari Laaksonen, Arja Halkoaho, Marja Kaunonen

**Affiliations:** ^1^Faculty of Social Sciences, Health Sciences, Tampere University, Tampere, Finland; ^2^School of Social Services and Health Care, Tampere University of Applied Sciences, Tampere, Finland; ^3^Applied Research Center, Tampere University of Applied Sciences, Tampere, Finland; ^4^Wellbeing Services County of Pirkanmaa, Tampere, Finland

**Keywords:** type 2 diabetes, risk, perception, inherited, family history, genomics

## Abstract

**Introduction:**

A family history is impacting the individual’s risk perception. The objective of this systematic review was to describe inherited risk perceptions of type 2 diabetes from the citizen’s viewpoint. The aim was to summarize and increase understanding so that the increased knowledge could be used effectively in type 2 diabetes risk communication in health care.

**Methods:**

We conducted a systematic review using CINAHL, Medline, and Scopus databases for hereditary, risk, perception, and diabetes related concepts, within the date range of 1.1.2017 to 2.8.2022. Eligible articles were English, peer-reviewed, and addressed the research question: how is hereditary risk of type 2 diabetes perceived? Returns were viewed independently by two authors, and evaluated using the appraisal criteria of the Joanna Briggs Institute. A thematic analysis was used for the synthesis of the data, yielding three themes describing perceptions of inherited risk in type 2 diabetes.

**Results:**

A total of 32 articles were included, of which 23 were quantitative, 5 qualitative, and 4 were mixed-methods studies. The extracted themes were (1) Identifying heredity as a risk factor, (2) Diversity of hereditary risk, and (3) Perception of the magnitude of personal risk.

**Discussion:**

The perception towards hereditary risk can vary from a desire to actively make a lifestyle change, to the view that diabetes is inevitable regardless of lifestyle. A positive family history increases the risk perception of type 2 diabetes, but the perceived magnitude of the risk may vary from person to person. The findings have the potential to be applied in healthcare’s risk communication.

## 1 Introduction

Diabetes prevalence has been predicted to increase worldwide from 10.5% in 2021 to 11.3% in 2030 and 12.2% in 2045, affecting an estimated 783 million people by that time. Another challenge is undiagnosed diabetes. In 2021, 44.7% (239.7 million) of people with diabetes were unaware of their condition (1). Attention should be paid to effective disease prevention, with an identification of those at risk and early diagnosis. Screening tools such as The Diabetes Risk Score can be used to identify individuals at high risk, with family history being one of the risk factors indicating an increased risk (2). However, merely identifying those at risk is insufficient; there must also be a deeper understanding of how individuals perceive their own risk. In previous research, increased perception of risk led to intentions to change health behavior. This effect was more pronounced when there was also a significant increase in self-efficacy and perceived severity (3). People with a higher familial risk of type 2 diabetes were also more likely to change behaviors to prevent diabetes, as well as to get tested and report diabetes diagnosis (4), but sometimes those with familial risk felt that they were unable to prevent diabetes (5).

Awareness of risk has been a significant part of health behavior theories. According to the Health Belief Model, weighting perceived susceptibility and perceived severity of illness or its sequelae (threat), perceived benefits of taking a particular action, and health motive (value of reduction of perceived threats) lead to health-related activities (6). Risk perception also plays a role in the initial motivation phase in The Health Action Process Approach (HAPA), but the risk perception alone is not sufficient to enable people to form an intention. However, with positive outcome expectancies and perceived self-efficacy, there might be a possibility to develop a health behavior intention and maintain actions through self-regulatory skills and strategies (7). Health literacy and the ability to make informed decisions for health and disease prevention also required an understanding of risk information, as well as the ability to interpret and evaluate one’s risk (8).

Dimensions and descriptions of risk perception varied among studies, and the concept of risk had not always been clearly described. However, a concept analysis of risk perception of developing diabetes included personal risk (perceived susceptibility or vulnerability), perceived severity, perceived likelihood, and an affective dimension (e.g., worry) (9). Walter et al. (10, 11) have explored familial risk perception, developing a model to understand how individuals with familial risk handle their vulnerability concerning common chronic diseases. Building on this, Daack-Hirsch et al. (12) investigated family risk in the context of type 2 diabetes. While some research has examined family history perception, there is limited study on genetic/genomic risk perception, despite the increasing use of genomic information. Consequently, the perception of family risk has evolved, gaining a new dimension with the incorporation of genomic risk perception. The content of the media coverage of the issue can be seen as indicative, at least to some extent, of people’s perception at that time, and looking further back, there was no mention of genetics or ethnicity in United Kingdom media in relation to diabetes risk in the early 1990s. Subsequently, in the early 2000s, ethnicity, race and culture began to be associated with diabetes (13).

Genetic risk for type 2 diabetes may have a similar impact on awareness than a positive family history of diabetes, but on the other hand, the nature of risk information differs from traditional family history risk. The nature of genetic risk information to type 2 diabetes was perceived as more reliable, realistic, and scientific than other available diabetes risk information (14). Although the risk assessment has numerical precision, its interpretation can be ambiguous. Despite its numerical nature, it is contextualized in family narratives (15).

The research question posed in this work is: how is the hereditary risk of type 2 diabetes perceived? The objective of this systematic review was to describe inherited risk perceptions of type 2 diabetes from the citizen’s viewpoint. The aim was to summarize and increase understanding so that the increased knowledge could be used effectively in type 2 diabetes risk communication in healthcare.

## 2 Methods

### 2.1 Search strategy and data sources

The systematic review covered a topic about heredity, risk and perception that can be worded in different ways. The context was type 2 diabetes, and the screening was focused on the citizens’ point of view. The diversity of concepts resulted in the following Boolean search phrase: (genom* OR genetic* OR polygenic OR famil* OR heredit* OR inherit* OR heritable) AND (“risk assessment” OR “risk analysis” OR “risk evaluation” OR “attitude to risk” OR “risk perception*” OR “risk score*” OR “risk factor*” OR predicti* OR predispos* OR “risk estimat*”) AND (knowledge or understand* or awareness or comprehen* or perception* or perceiv* or attitude* or experience* or interpret*) AND (diabetes or diabetic).

The same search phrase was used in CINAHL Complete, Medline, and Scopus databases, where publication date of 1.1.2017–2.8.2022, English language and peer reviewed articles were imposed as database limitations (Table 1). “Title-abs-key” was also added to the Scopus search to consider the search phrase only in the title, abstract or keywords. An information specialist was utilized in formulating the search strategy to ensure the functionality of the search from different databases. The systematic review followed the PRISMA flow-chart for database and records screening (16).

**Table 1:** Inclusion and exclusion criteria employed during the database search and screening process.

Inclusion criteria	Exclusion criteria
**Database limiters** Publication date 1.1.2017–2.8.2022	
English language	
Peer reviewed	
**Quality appraisal** > 50% of JBI criteria	**Quality appraisal** ≤ 50% of JBI criteria
**Inclusion during screening:** Citizens’ perception or understanding of the genetic/genomic risk or familial/hereditary risk of type 2 diabetes	**Exclusion during screening:** A healthcare professional’s perspective as a professional
If the title or abstract mentioned the risk perception, but not yet specified genetic or familial risk, records were read in more detail to determine suitability.	After being read in more detail, if the risk perception was not related to genetic or familial risk, records were excluded.
If the title or abstract mentioned multifactorial diseases/common complex diseases in general, records were read in more detail to determine suitability.	After being read in more detail, multifactorial diseases/common complex diseases in which type 2 diabetes could not be specified or interpreted separately in the results were excluded.

### 2.2 Study selection and quality appraisal

**Figure 1:**
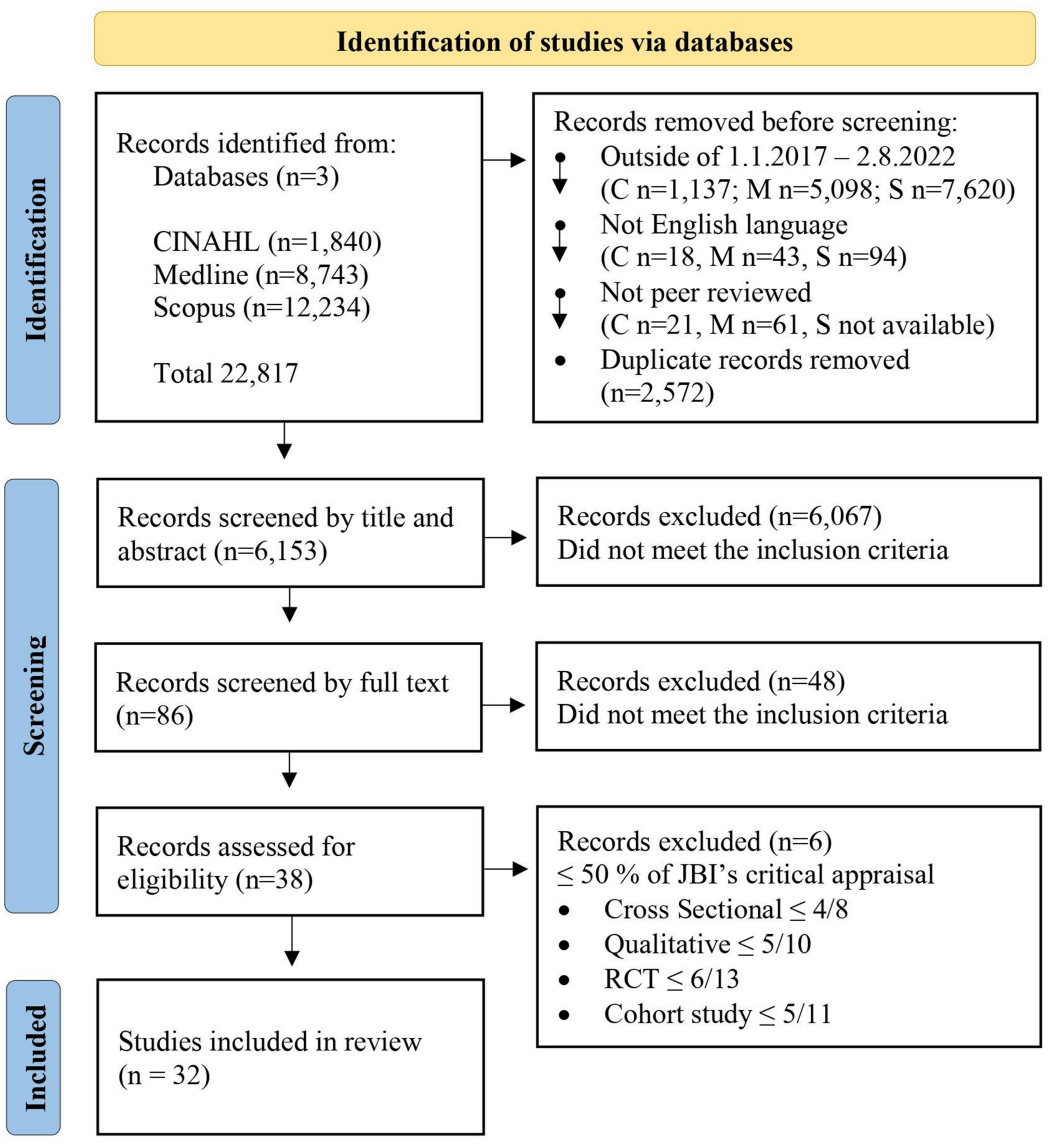
PRISMA flow-chart for database and records screening.

The records were selected independently by the first two authors using Covidence (RRID:SCR_016484), first at the title and abstract level, and finally at the full text level (Figure 1). When using the Covidence program, it is not possible to initially select records only based on the title alone, so both the title and the abstract needed to be read in the first round. Any records where authors had different opinions were discussed, in order to find a unified solution for the selection. If the exclusion criteria did not appear in the title or abstract, but there was also no certainty of inclusion, the records were retained to be read as full text.

All of the selected full-text records were evaluated independently by the first two authors using the appraisal criteria of the Joanna Briggs Institute (JBI) (17). We used JBI checklists for Analytical Cross Sectional Studies (*n* = 19), Qualitative Research (*n* = 12), Randomized Controlled Trials (*n* = 2), and Cohort Study (*n* = 1). In the case of mixed methods (*n* = 4), we used both Cross Sectional and Qualitative Research appraisal criteria, or only criteria of the study type from which the data was obtained. When there was a difference in the appraisal between two authors, the studies were reviewed again, and a consensus was reached. We required scores above 50% for the record to be accepted as part of this systematic review. If the records did not satisfactorily fulfill the appraisal criteria, it was excluded at this stage. From the selected records, the authors, year of publication, country, method, sample, and results related to the themes were listed (see Table 2).

**Table 2:** Information and specific items from selected articles in the systematic review.

Ref. no.	Author, year of publication	Objective of the study	Study method, and sample	The results related to the themes of the review	Quality assessment: JBI’s critical appraisal
12	Daack-Hirsch et al. (2018) United States	To extend and enrich the FRP model for type 2 diabetes (T2D) by exploring the risk-personalization process with a more diverse group of participants who had a positive family history of T2D.	Mixed methods: qualitative arm of the study Semi structured interviews Purposeful sampling, *n* = 113, 18–60 years, who had a positive family history of diabetes, and did not have diabetes	Identifying heredity as a risk factor: family history was one of the main risk factors and a risk factor alongside health behavior risk. Causal explanations were a combination of genes and behavior. Those with a moderate familial risk emphasized behavioral factors over genetics compared with those who had a high-familial risk. Family history: family history was recognized when several relatives and multiple generations had type 2 diabetes. Recognized through paternal lineage or both sides of the family. The terms describing family history were interchangeable. Genetic/genomic risk: genetics was mentioned as an inherited risk, but also as a personal risk factor that is not necessarily inherited. Genetics was described using terms that could not applied in the specific context of type 2 diabetes. Ethnic risk: some genetic characteristics were seen to increase the risk of type 2 diabetes, such as race, and certain ethnic groups were seen as more prone to type 2 diabetes than others. Inherited cultural and health behavior: “Running in the family” also meant health behaviors, weight, and culture. Those who were moderate- and high-risk families talked more about health behavior risk factors than genetics as “running in their family.”	Qualitative research 8/10
19	Kharono et al. (2017) Uganda	To assess the knowledge, attitudes, and risk perceptions of university medical students in Uganda regarding diabetes mellitus.	Quantitative, descriptive cross-sectional study Self-administered semi-structured questionnaires Simple randomization, *n* = 378, 18–40 years	Identifying heredity as a risk factor: family history was one of the main risk factors.	Analytical Cross Sectional Study 6/8
20	Al-Thani et al. (2018) Qatar	To examine the community diabetes knowledge, perceptions, and awareness among the public in Qatar regarding disease symptoms, risk factors, complications, prevention, and associated behaviors.	Quantitative study Face-to-face interview, semi-structured questionnaire Purposive sampling, *n* = 501, > 16 years	Identifying heredity as a risk factor: family history was one of the main risk factors and a risk factor alongside health behavior risk.	Analytical Cross Sectional Study 8/8
21	Yang et al. (2018) United States	To identify differences in perceived risk for diabetes and/or prediabetes between different racial and/or ethnic populations and to examine associations between perceived risk and actual risk among racial and/or ethnic groups using a nationally representative sample.	Quantitative, cross-sectional observational study Interviews and physical examinations Stratified, multistage probability sampling, *n* = 10,999, ≥ 20 years	Identifying heredity as a risk factor: family history was one of the main risk factors and a risk factor alongside health behavior risk. Ethnic risk: there were no significant differences between ethnic groups in the agreement between actual and perceived risks, but Non-Hispanic Black and Hispanic populations perceived their risk more when they had a poor diet. Increased risk perception: a family history was associated with increased perceived risk.	Analytical cross sectional study 7/8
22	Cuschieri et al. (2019) Malta	To determine the level of diabetes awareness among a high-risk dysglycaemic population in relation to socio-demographic, lifestyle and family history of diabetes as well as to body mass index (BMI) and blood pressure measurements.	Quantitative, cross-sectional study Questionnaire *n* = 155, 20–70 years, who had impaired fasting blood glucose (5.60–6.99 mmol/L)	Identifying heredity as a risk factor: family history was one of the main risk factors.	Analytical Cross Sectional Study 7/8
23	Ard et al. (2020) United States	To understand if a family history of T2DM played an influential role in individuals making positive health behavior changes for T2DM prevention.	Qualitative study Face-to-face or online interviews Purposive sampling, *n* = 20, ≥ 18 years, with a family history of T2DM, but had not been diagnosed with the disease	Identifying heredity as a risk factor: family history was one of the main risk factors and a risk factor alongside health behavior risk. Risk in one’s own family: the perception of risk was experienced by the significant events. Family history: family history had an influence on being more aware of health behaviors.	Qualitative research 8/10
24	Daack-Hirsch et al. (2020) United States	To characterize two key concepts, salience and vulnerability, within the familial risk perception (FRP) model among unaffected individuals, at increased familial risk for T2D.	Mixed methods: qualitative and quantitative, qualitative arm primary focus Semi-structured interviews and questionnaire Purposeful sampling, qual. *n* = 111, quant. *n* = 153, 18–60 years, who had a positive family history of diabetes, and did not have diabetes	Risk in one’s own family: the perception of risk was experienced by the significant events. While processing one’s own risk, health behaviors, body type and age at the time of diagnosis were compared with those of family members who had type 2 diabetes to formulate a personal risk. Identifying heredity as a risk factor: family history was one of the main risk factors. Perception of the magnitude of personal risk: people used cognitive strategies to explain their risk value. Worry was eased by the knowledge that type 2 diabetes was a manageable and ‘not as life-threatening’ disease. The accuracy of the risk perception: most participants underestimated their overall risk.	Qualitative Research 8/10
25	Sharma et al. (2020) India	To assess the knowledge, and awareness about diabetes and its complications among different strata of people attending a tertiary care eye institute in north India.	Quantitative, cross sectional survey Personal interviews Random sampling, *n* = 530, > 18 years	Identifying heredity as a risk factor: family history was one of the main risk factors and a risk factor alongside health behavior risk.	Analytical Cross Sectional Study 5/8
26	Shiferaw et al. (2020) Ethiopia	To assess knowledge and risk perceptions towards diabetes mellitus and its associated factors among Debre Berhan community members, in northeast Ethiopia.	Quantitative, cross-sectional study Questionnaire through face-to-face interviews Three kebeles were selected by the lottery method, systematic random sampling to select the study unit among households, *n* = 423, ≥ 18 years	Identifying heredity as a risk factor: family history was one of the main risk factors and a risk factor alongside health behavior risk.	Analytical Cross Sectional Study 6/8
27	Alharthi et al. (2021) Saudi Arabia	To assess diabetes mellitus risk factors (DM-RFs) awareness among adults residing in Saudi Arabia.	Quantitative, cross-sectional study Questionnaire Random sampling, *n* = 404, 18–65 years	Identifying heredity as a risk factor: family history was one of the main risk factors.	Analytical Cross Sectional Study 6/8
28	Anyanti et al. (2021) Nigeria	To assess levels of awareness, knowledge, attitude and practices relating to hypertension and diabetes among adults aged 35 years resident in selected communities in Nigeria prior to the commencement of project interventions.	Quantitative, descriptive cross-sectional study Interviewer-administered, semi-structured questionnaire Multi-stage sampling, *n* = 824, ≥ 35 years	Identifying heredity as a risk factor: family history was one of the main risk factors and a risk factor alongside health behavior risk.	Analytical Cross Sectional Study 7/8
29	Guo et al. (2019) China	(1) To describe the perceived risk of T2DM for mothers of preschool children in China; (2) to identify the actual risk of developing T2DM for mothers of preschool children; and (3) to explore the factors associated with the perceived T2DM risk for mothers of preschool children.	Quantitative, multisite, cross-sectional study Self-report questionnaire Convenience sampling, *n* = 176 mothers without diabetes	Identifying heredity as a risk factor: family history was identified as a risk factor alongside health behavior risk. Those with a higher family history risk also had more knowledge about diabetes risk. Increased risk perception: a family history was associated with an increased perceived risk.	Analytical Cross Sectional Study 6/8
30	Cunningham et al. (2020) United States	To report exploratory qualitative findings on the perceptions of diabetes family history and experiences on the illness representations of individuals with diabetes. Note: the results do not differentiate between type 1 and type 2 diabetes. Type 2 diabetes was interpreted, among other things, through authentic quotes.	Qualitative, exploratory study Self-reported demographic data, electronic medical record reviews, open-ended semi-structured interviews Convenience sample, *n* = 89, ≥ 18 years, with type 1 or type 2 diabetes	Identifying heredity as a risk factor: family history was identified as a risk factor alongside health behavior risk. Causal explanations were a combination of genes and behavior. Risk in one’s own family: witnessing family members’ complications motivated behavior change. The progression of the disease in previous generations was not necessarily seen as recurring. They had access to preventive health information that previous generations did not have. Family history: family history included aunts, uncles, and siblings. Diabetes was perceived as inevitable. Inherited cultural and health behavior: health behaviors were perceived as hereditary.	Qualitative Research 7/10
31	Teh et al. (2021) Singapore	To explore the postpartum dietary and physical activity-related beliefs and behaviors among women in Singapore who had GDM in their most recent pregnancies.	Qualitative, descriptive research design Semi-structured in-depth interviews Purposive sampling, *n* = 14, ≥ 21 years, with self-reported history of GDM	Identifying heredity as a risk factor: a positive family history was also perceived to be a more significant risk factor of developing type 2 diabetes than a personal history of gestational diabetes.	Qualitative Research 9/10
32	Pelullo et al. (2019) Italy	To evaluate knowledge about diabetes; to assess the perception of risk for developing diabetes; and to determine the factors associated with these outcomes of interest.	Quantitative, cross-sectional study Self-administered questionnaire Six schools randomly selected, the students selected through a simple random sampling, a random sample of parents, cluster sampling, *n* = 527 parents	Identifying heredity as a risk factor: those with a higher family history risk also had more knowledge about diabetes risk. Increased risk perception: a family history was associated with increased perceived risk.	Analytical Cross Sectional Study 6/8
33	Daack-Hirsch et al. (2019) United States	To explore patterns in the previously coded data set to identify possible FRP (familial risk perception) subtypes.	Mixed methods: previously collected interviews and survey data. Qualitative and quantitative data combined in cluster analysis. Clusters analyzed quantitatively. Purposeful sampling, *n* = 109, 18–60 years, who had a positive family history of diabetes, and did not have diabetes	Identifying heredity as a risk factor: causal explanations were a combination of genes and behavior. Those who most emphasized genetic risk as the causal explanation worried more about developing type 2 diabetes. Diabetes was seen as a preventable disease, and the risk of diabetes was seen as modifiable. Inherited cultural and health behavior: both genetic and nongenetic factors were seen as being transmitted within the family. Increased risk perception: those who emphasized genetic risk to be a causal explanation more than a behavioral risk assessed their risk higher. Those who were not clear about the genetic factors assessed their risk at the lowest.	Analytical Cross Sectional Study 7/8 Qualitative Research 6/10
34	Fiallos et al. (2021) United States	To describe the lay beliefs of disease inheritance held by Spanish-speaking members of the US Latina immigrant population.	Qualitative study Semi-structured interviews, in-person interviews Purposive sampling, *n* = 20, > 18 years	Identifying heredity as a risk factor: women more generally believed that they could modify health behavior to prevent or control diabetes. Diabetes was seen as a preventable disease, and the risk of diabetes was seen as modifiable. Risk in one’s own family: they had access to preventive health information that previous generations did not have. Family history: diabetes was seeing running in the family. Family history had an influence on being more aware of health behaviors. Genetic/genomic risk: diabetes was seemed to be the most salient genetic condition. Knowing about genetics and family made it possible to prepare for better health behavior. Some participants knew about genetic risk tests that could be used to find out the assessment of genetic risk. Diabetes (as a genetic disease) was seen a preventable by eating healthily and doing more exercise.	Qualitative Research 9/10
35	Faletau et al. (2020) New Zealand	To develop an understanding of how being ‘at risk’ of developing type 2 diabetes is perceived by Tongan people with prediabetes living in Auckland, New Zealand.	Qualitative study One-on-one, semi-structured interviews Purposeful sampling, *n* = 12, 20–59 years, who had prediabetes	Risk in one’s own family: the perception of risk was experienced by the significant events. Prediabetes and diagnosed type 2 diabetes could not always be distinguished. Family history: if there was no family history at all, there was a disbelief in the possibility of getting diabetes.	Qualitative research 10/10
36	Grabowski and Andersen (2020) Denmark	To explore barriers to prevention in families with at least one adult with type 2 diabetes.	Qualitative study Semi-structured workshops groups *n* = 26, 50–75 years, people with type 2 diabetes and *n* = 31 their relatives	Risk in one’s own family: the role and significance of diabetes in the family unit affected how diabetes was perceived, managed, and prevented in the family. When diabetes directly affected a family member, motivation for diabetes management was often higher. Families worried about their offspring’s health behavior and the development of type 2 diabetes. Inherited cultural and health behavior: families inheriting an unhealthy lifestyle left very little room for making decisions about changing health behaviors. Awareness rarely transformed into actual preventive action.	Qualitative research 9/10
37	Khlaifat et al. (2020) Jordan	To assess diabetes knowledge, risk perception and practice among diabetes-free university students in South Jordan.	Quantitative, exploratory cross-sectional study Self-administered structured questionnaire Convenience sample, *n* = 2,158, 18–50 years	Risk in one’s own family: caring for a relative was associated strongly with practice levels, but family history alone was negatively associated with perception and practice levels.	Analytical Cross Sectional Study 5/8
38	Badlishah-Sham et al. (2020) Malaysia	To (1) determine the distribution of type 2 diabetes patients regarding their willingness to accept training to speak to their offspring, (2) determine the distribution of type 2 diabetes patients regarding their willingness to accept training based on the HBM and (3) to determine the factors associated with their willingness to accept training.	Quantitative, cross-sectional study Self-filling questionnaires, socio-demographic characteristics collected via face-to-face interview Convenience sampling, *n* = 425, ≥ 18 years, type 2 diabetes patients who had at least one offspring without type 2 diabetes	Risk in one’s own family: willingness to speak about their type 2 diabetes with the offspring. The perception of the probability of the disease and a concern that the offspring will develop type 2 diabetes increased the willingness to discuss.	Analytical Cross Sectional Study 7/8
39	Vaja et al. (2021) United Kingdom	To generate a grounded theory (GT) to understand how SA individuals create and construct the meaning of T2D prevention and how this meaning influences their lived behaviours.	Qualitative study One-to-one semi-structured interviews Snowball sampling, *n* = 20, 25–62 years, without a diagnosis of T2D	Family history: diabetes was perceived as inevitable. Genetic/genomic risk: personal risk was considered as being hereditary. Ethnic risk: traditional and cultural practices were seen to be hereditary, in which restricted or prohibited the perceived possibilities for change.	Qualitative research 10/10
40	Rego et al. (2019) United States	To address the question of the population’s motivations for undergoing exome sequencing, their expectations, reactions, and perceptions of utility. Note: genome test results were not always clear targeting type 2 diabetes, as the test was for multifactorial diseases. Attempted to take only type 2 diabetes into account.	Qualitative study In-depth semi-structured interviews Recruited from on going study, *n* = 12, 45–74 years	Genetic/genomic risk: the reason to participate in multi-omics studies and get exome results was the intention to be proactive and a desire to know the genetic underpinnings of those who already had type 2 diabetes. Particularly, the genome test was often used to bring closure and understanding to an already existing condition. Very few reported making any changes to their lifestyle due to genomic results. Genome test results strengthened the intention for pursuing a healthier lifestyle, even if it had already been attempted before.	Qualitative Research 8/10
41	Charbonneau et al. (2020) United States, United Kingdom, Japan, Australia	To assess comprehension and psychological and behavioural reactions to hypothetical DTCGT reports that varied according to the type of test (type 2 diabetes, colorectal cancer, drug sensitivity), severity of risk, lifestyle/family history information and validity of genetic results.	Quantitative, cross-sectional study Experimental design: pre-scenario questions, scenario directed questions, pro-scenario questions. Sampling by country, gender, age quotas, US (*n* = 1,000), UK (*n* = 1,014), Japanese (*n* = 1,018) and Australian (*n* = 1,000), 18–91 years	Genetic/genomic risk: participants reported significantly more likely they would not make decisions if the risk was low than when the risk was high.	Analytical Cross Sectional Study 5/8
42	Joiner et al. (2022) United States	To determine whether perceived risk for diabetes differs by race and ethnicity among a nationally representative sample of U.S. adults with undiagnosed prediabetes.	Quantitative, cross-sectional study, series of multistage probability surveys Purposive sample from those who participated in the NHANES, *n* = 4,005, ≥ 20 years with undiagnosed prediabetes	Ethnic risk: there were no statistically significant associations between ethnicity and perceived risk for diabetes. But in adjusted analyses, there were some differences, and Hispanic and Non-Hispanic Black were associated with a higher likelihood of reporting no perceived risk of type 2 diabetes compared to non-Hispanic white. Increased risk perception: a family history was associated with increased perceived risk.	Analytical Cross Sectional Study 8/8
43	de la Haye et al. (2021) United States	To evaluate the acceptability and usability of an evidence-based Family health history FHH tool for use in community settings in an under-resourced, African American community.	Mixed-methods: quantitative and qualitative Baseline, 6 weeks intervention, focus groups Purposive sample, *n* = 62 baseline, *n* = 51 follow-up, *n* = 10 in two focus groups, 30–72 years	Perception of the magnitude of personal risk: personalized pedigree and disease risk in the community were more salient than the level of risk information (average or increased). Accuracy of the risk perception: perception was towards a more consistent or underestimation of risk perceptions rather than overestimation.	Analytical Cross Sectional Study 6/8 Qualitative research 6/10
44	Halmesvaara et al. (2022) Finland	To confirm earlier findings concerning risk perception and self-efficacy and expand from previous studies by investigating the emotional reaction to the test results.	Quantitative, randomized controlled trial Pre-survey, risk estimate and post-survey Random sample, *n* = 1,368 (experimental *n* = 714, control group *n* = 649)	Perception of the magnitude of personal risk: participants with low risk felt more in control than participants with very high risk. Those with low risk were significantly less worried than participants who had a higher risk for type 2 diabetes, but there was no statistical significance of the type of risk given. Accuracy of the risk perception: those participants who were at low risk perceived their risk to be lower compared with those who were at higher risk levels, but there were no significant differences for the type of risk information given.	Randomized controlled trials 8/13
45	Wu et al. (2017) United States	To determine the impact of type 2 diabetes family health history (FHH) and genetic risk counseling on behavior and its cognitive precursors.	Quantitative, randomized controlled trial (RCT) design Baseline surveys, intervention, surveys at 3 and 12 months Convenience sample, randomized to two groups, *n* = 391, (intervention *N* = 198, control *N* = 93), at 3 months *n* = 368 (*N* = 170/198), at 12 months *n* = 358 (*N* = 160/198), age 18–81 years, no self-reported history of diabetes	Perception of the magnitude of personal risk: participants had an overall strong perception of personal control over type 2 diabetes risk, which did not vary on family history risk or genetic risk levels. Increased risk perception: a family history was associated with increased perceived risk. Those who had more genetic risk were more alleles likely to perceive a more serious risk for type 2 diabetes. This was strongest with those who had an average and moderate family history, but those with a high family history risk had no statistically significant changes in risk perception.	Randomized controlled trials 9/13
46	Heidemann et al. (2019) Germany	To evaluate perceived diabetes risk in comparison to actual diabetes risk in a representative sample of the general adult population and to investigate whether sociodemographic and diabetes risk factors as well as healthcare and psychological factors contribute to explain diabetes risk perception in the subgroup of adults at high actual diabetes risk.	Quantitative study Telephone interview survey Random sampling and a larger subsample for diagnosed diabetes, *n* = 3,806 (*n* = 2,327 without diabetes, *n* = 639 with actual diabetes risk), 18–97 years	Increased risk perception: a family history was associated with increased perceived risk. Accuracy of the risk perception: those who had an elevated or high actual diabetes risk and perceived themselves at increased (i.e., moderate or high) diabetes risk were found to have a significant association with a family history of diabetes	Analytical Cross Sectional Study 6/8
47	Khan et al. (2022) United States	To determine diabetes knowledge and future disease risk perception among college students in a large public university in West Virginia, a state entirely within the Appalachian region.	Quantitative, cross-sectional survey Online survey Purposive sample, *n* = 697, ≥18 years	Increased risk perception: a family history was associated with increased perceived risk.	Analytical Cross Sectional Study 6/8
48	Antwi et al. (2020) United States	To generate preliminary data on the perception of T2D and further determined the prevalence of T2D risk factors among college students at an upstate New York campus.	Quantitative, cross-sectional study (pilot) Online survey questionnaires, anthropometric and metabolic profile. Purposive sampling, *n* = 44, ≥ 18 years, non-diabetic college (*n* = 132 submitted online questionnaire)	Increased risk perception: no effect on the perceived seriousness of the disease.	Analytical Cross Sectional Study 7/8
49	Silarova et al. (2018) United Kingdom	To identify the proportion of individuals with an accurate perception of their risk of type 2 diabetes (T2D) prior to, immediately after and 8 weeks after receiving a personalised risk estimate. To explore what factors are associated with underestimation and overestimation immediately post-intervention.	Quantitative, cohort study Questionnaires: baseline, post-intervention, 8 weeks after intervention Data collected form the diabetes risk communication trial, *n* = 373 baseline, *n* = 365 after intervention, *n* = 368 8 weeks after intervention	Accuracy of the risk perception: people tend to overestimate rather than underestimate their risk. Those who overestimated their T2D risk continued to overestimate the risk. Those who underestimated their T2D risk perceived their risk more accurately after receiving a risk estimate. Those who were actually at higher risk and received a higher risk estimate were more likely to underestimate their risk. Participants received a genotypic or phenotypic risk estimate for type 2 diabetes, but there were no differences in risk perception between these risk information types.	Cohort study 8/11

### 2.3 Data analysis

Thematic analysis was used for the data synthesis. Even though the research question was about *perception* bringing up experience or understanding, we used a critical approach (18) since review data was interpreted as being a secondary source and included both qualitative and quantitative studies. Perceptions of pure experientiality cannot be achieved, but both data types strengthened and shaped the created themes well throughout the analysis. However, the qualitative data was coded first, followed by the quantitative data. If the study in question used a mixed method, the qualitative component was coded first.

The data coding in the review was a systematic process. The codes were representative of meanings that provided responses to the research question: how is the hereditary risk of type 2 diabetes perceived? The analysis resulted in the identification of three themes. The data were checked twice for coding to ensure that all meanings were included. A second check also strengthened the understanding of the whole data. Although a few initial themes began to form in the coding phase, the main themes were actively developed only after coding process and a good familiarization with the data had been achieved. As a further element, interpretation was used as a narrative beyond the data to make sense of the theme in its context (18).

## 3 Results

The literature search included a total of 22,817 records from three databases (CINAHL, Medline, Scopus), and after database limitations and duplications were removed, 6,153 records were left for title and abstract screening, 86 records for full-text screening, and 38 records underwent a quality assessment. Finally, 32 records were included in the systematic review (Figure 1). Inherited risk perceptions were described through three themes: (1) Identifying heredity as a risk factor, (2) Diversity of hereditary risk, and (3) Perception of the magnitude of personal risk.

The studies featured in this review were drawn from 21 different countries (Table 2), with 13 from the United States, two from the United Kingdom, and single studies from China, Denmark, Ethiopia, Finland, Germany, India, Italy, Jordan, Malaysia, Malta, New Zealand, Nigeria, Qatar, Saudi Arabia, Singapore, and Uganda. One study gathered data from the United States, the United Kingdom, Japan, and Australia. In addition, some studies focused on immigrants, such as Latino immigrants in the United States or South Asians in the United Kingdom.

### 3.1 Identifying heredity as a risk factor

Perception the hereditary risk of type 2 diabetes can begin simply by recognizing it as a risk factor. Here, the terms family history, genetics, and heredity are all combined into the same risk factor concept, describing inherited risk among other identified risk factors. The view is also taken that there is no reason to separate family history and genetics, although they will be described separately later in a different context.

A family history of diabetes was identified as one of the main risk factors for type 2 diabetes (12, 19–28), and as being a risk factor alongside health behavior risks (12, 20, 21, 23, 25, 26, 28–30). A positive family history was also perceived to be a more significant risk factor of developing type 2 diabetes than a personal history of gestational diabetes (31). Those with a higher family history risk also had more knowledge about diabetes risk (29, 32).

As a multifactorial disease, causal explanations were perceived as a combination and interaction of genes and behavior (12, 30, 33), but the emphasis on these explanations varied between people (12, 33). Those who most emphasized genetic risk as the causal explanation worried more about developing type 2 diabetes, while those who were not clear about the genetic factors more likely perceived that type 2 diabetes is not heritable and were less concerned about developing type 2 diabetes. However, weighting explanations between genetics and behaviors did not affect health behaviors (33). Aging was generally seen to increase diabetes risk depending on genetic background and health behavior. Those with a moderate familial risk emphasized behavioral factors over genetics compared with those who had a high-familial risk, and women emphasized behavioral factors over genetics compared with men (12). Also, women more generally believed that they could modify health behavior to prevent or control diabetes (34). Although family history was generally seen as a major risk factor, type 2 diabetes was seen as a preventable disease, and the risk of diabetes was seen as modifiable (33, 34).

### 3.2 Diversity of hereditary risk

#### 3.2.1 Risk in one’s own family

A family is a unit where diabetes becomes salient and where one’s risk perception of type 2 diabetes is processed. The perception of risk was experienced by the significant events when a family member received a diagnosis, experienced diabetes management, or witnessed complications. Those events were turning points for a close family member to reflect concern about their own risk, the seriousness of the disease, and its consequences (23, 24, 35). Since understanding was constructed upon experiences, for example, prediabetes and diagnosed type 2 diabetes could not always be distinguished (35).

The role and significance of diabetes in the family unit affected how diabetes was perceived, managed, and prevented in the family. When diabetes was isolated only to the person with diabetes, it reduced intra-familial involvement and prevention. Families had collective practices that sustained unhealthy habits. These practices were also passed down to the next generation. When type 2 diabetes was not perceived as significant, it was difficult to create collective practices for the daily management of diabetes and change health behavior as an individual within the family. Prevention required a sense of significance and an imagination of the family’s future (36).

The family member’s closeness with a person with diabetes played a role in risk perception. When diabetes directly affected a family member, motivation for diabetes management was often higher (36). Witnessing family members’ complications motivated behavior change (30). Also, caring for a relative was associated strongly with practice levels. Interestingly however, in this study family history alone was negatively associated with perception and practice levels (37).

While processing one’s own risk, health behaviors, body type and age at the time of diagnosis were compared with those of family members who had type 2 diabetes to formulate a personal risk (24). The goal was to avoid a similar situation and make a change. The progression of the disease in previous generations was not necessarily seen as recurring, because now treatment was sought earlier (30) and they had access to preventive health information that previous generations did not have (30, 34).

In the family unit, the perception of risk can be processed by experiencing and comparing the situation with the family member who has diabetes. Furthermore, those with type 2 diabetes perceived a risk for their children as well. Families worried about their offspring’s health behavior and the development of type 2 diabetes, but youth were often seen as unreachable to health behavior change in risk communication. The lack of diabetes communication could be due to a lack of knowledge, but often also as a lack of perceived seriousness. The parent’s inability to communicate risk effectively confirms the offspring’s perception that future health problems are not really concerns (36). However, almost two-thirds were willing to speak about their type 2 diabetes to their offspring, and those with a family history of type 2 diabetes were more willing. The perception of the probability of the disease and a concern that the offspring will develop type 2 diabetes increased the willingness to discuss the topic, but the perceived severity was not found to be significant (38).

#### 3.2.2 Family history

In addition to the risk dealt with within one’s own family, hereditary risk can be considered as a more general family history risk. Also, family history is often featured as a background factor in research, and in that context, it is not always possible to say how family history is understood or how close a relationship the participant had with the family member who had type 2 diabetes.

Family history was recognized when several relatives and multiple generations had type 2 diabetes (12) including aunts, uncles, and siblings (30), or just simply seeing diabetes running in the family (34). It could be recognized through paternal lineage or on both sides of the family. But if there was only one affected person (12) or no family history at all, there was a disbelief in the possibility or likelihood of getting diabetes (35). The terms describing family history were interchangeable. Family history, running in the family, genetics, and inherited could all mean the same thing (12).

Information about the family’s history was handled differently regarding the possibility of prevention. Family history had an influence on being more aware of health behaviors (23, 34), but diabetes was also perceived as inevitable due to family history (30, 39). In this context, inevitable meant, for example, that the risk could not be changed even by healthy eating (39).

#### 3.2.3 Genetic/genomic risk

Genetic risk, as we define it here, refers to the risk perception obtained through genetic testing or an understanding of type 2 diabetes genetics, distinct from the colloquial notion of it being solely a matter of family tradition.

Diabetes was frequently mentioned as a genetic disease, which seemed to be the most salient genetic condition. The reason diabetes has not been categorized as a genetic disease was because it was not seen as a rare disease. But in both cases, if diabetes was believed to be either a genetic or non-genetic disease, the risk was believed to be modifiable. Knowing about genetics and family made it possible to prepare for better health behavior (34). Genes were seen to interact with other risk factors and predispose to the development of diabetes. Predisposition meant either increasing the risk of a family member having type 2 diabetes, or having the gene or genotype that predisposes diabetes. Genetics was mentioned as an inherited risk, but also as a personal risk factor that is not necessarily inherited (12). On the other hand, elsewhere, personal risk was considered as being hereditary (39). Differences in perception can be explained, among other things, by a lack of understanding of genetics and concepts, and while sometimes the role of genetics was described using terms that could apply to Mendelian inheritance but not applied in the specific context of type 2 diabetes (12).

Some participants knew about genetic risk tests that could be used to find out the assessment of genetic risk (34). The reason to participate in multi-omics studies and get exome results was the intention to be proactive and a desire to know the genetic underpinnings of those who already had type 2 diabetes. Particularly, the genome test was often used to bring closure and understanding to an already existing condition (40).

As mentioned earlier, the family history did not increase the practice levels (37), and the same was seen with genetic risk. Although diabetes (as a genetic disease) was seen a preventable by eating healthily and doing more exercise (34), very few reported making any changes to their lifestyle due to genomic results. However, genome test results strengthened the intention for pursuing a healthier lifestyle, even if it had already been attempted before (40). Also, when participants received direct-to-consumer genetic test scenarios, they significantly reported that it was more likely that they would not make decisions if the risk was low than when the risk was high (41). However, it should be noted that the decisions made by the participants in this study were only intentions, as there were no actual personal genomic results.

#### 3.2.4 Ethnic risk

Some genetic characteristics were seen to increase the risk of type 2 diabetes, such as race, and certain ethnic groups were seen as more prone to type 2 diabetes than others (12). Type 2 diabetes was seen as an inevitable and accepted social norm due to ethnicity in the community. Such a perception of preordained destiny led to a reduced sense of responsibility and of value in assessing type 2 diabetes risk, as well as the decreased perceived ability to reduce risk. Also, traditional and cultural practices were seen to be hereditary, in which restricted or prohibited the perceived possibilities for change (39). In the diversity of hereditary risk, ethnic risk perception is therefore very different from the risk in one’s own family and when the salience of diabetes arouses the desire to make a change in lifestyle. However, Joiner et al. (42) brought up that there were no statistically significant associations between ethnicity and perceived risk for diabetes. But in adjusted analyses, there were some differences, and Hispanic and Non-Hispanic Black were associated with a higher likelihood of reporting no perceived risk of type 2 diabetes compared to Non-Hispanic White. Yang et al. (21) also found that there were no significant differences between ethnic groups in the agreement between actual and perceived risks, but Non-Hispanic Black and Hispanic populations perceived their risk more when they had a poor diet.

#### 3.2.5 Inherited cultural and health behavior

The extent of the diversity of hereditary risk is illustrated by the heritability of culture and health behaviors which are passed down from one generation to another, increasing the risk of type 2 diabetes. Both genetic and nongenetic factors were seen as being transmitted within the family (33). Health behaviors were perceived as hereditary, similar to how diabetes was seen as hereditary (30). The concept of “running in the family” also meant more than just genetics, and reflected health behaviors, weight, and culture. Those who were moderate- and high-risk families talked more about health behavior risk factors than genetics as “running in their family” (12). Hereditary lifestyle risks were also mentioned, where families inheriting an unhealthy lifestyle left very little room for making decisions about changing health behaviors, both as individuals within the family and as a family among other families. Awareness rarely transformed into actual preventive action (36), and similar to this was the concept of ethnic risk perception where the strong socio-cultural importance of the family and traditional food practices were reasons for not being able to change lifestyle (39).

**Figure 2:**
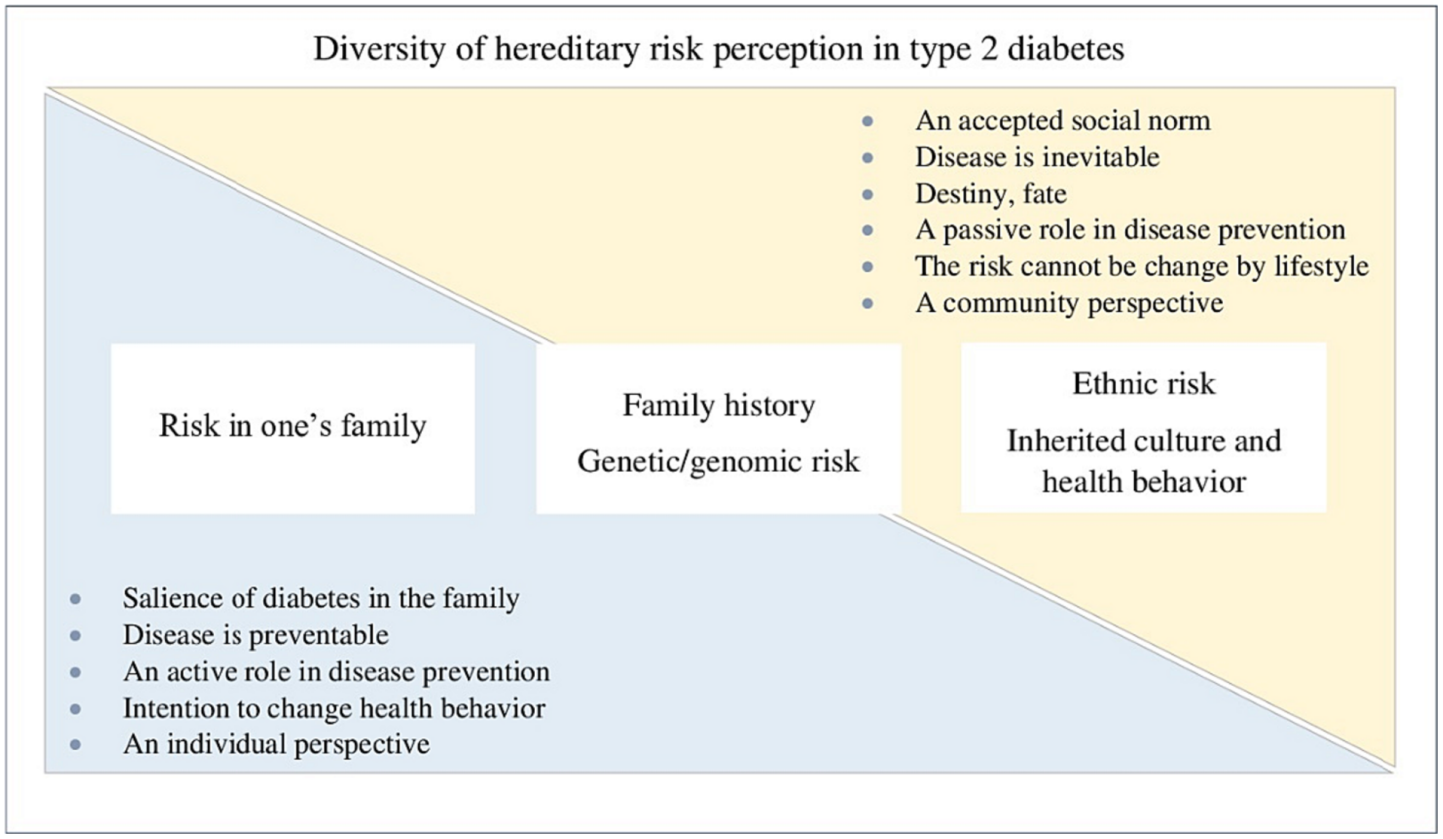
Indicative description of hereditary risk types in type 2 diabetes and the ensuing perceptions.

Figure 2 offers a summary indicative description of the diversity of hereditary risk perceptions. The figure should not be interpreted in such a straightforward manner, but is intended to give direction to various inherited risk perceptions and the factors influencing them. For example, risk in one’s own family can increase prevention intentions through the disease experiences of close relatives, but also lead to a perception of the disease as being inevitable if diabetes remains more superficial and distant. In the figure, a different type of inherited risk can be more to the right or to the left, depending on the individual’s perception of risk.

### 3.3 Perception of the magnitude of personal risk

This theme describes the perception of the magnitude of personal risk and its formation. The perception of risk was based on how participants interpreted their personalized pedigree and understood the disease risk in their community. Personalized pedigree and disease risk in the community were more salient than the level of risk information (average or increased) (43). People used cognitive strategies to explain their risk value. Also, they combined several risk factors to assess their personal risk, such as controllable lifestyle habits and uncontrollable risks like genetics and family history, where the risk was assessed to be low despite a familial risk when the family history was balanced with behaviors (24). Participants with low risk felt more in control than participants with very high risk (44). However, there was an overall strong perception of personal control over type 2 diabetes risk, which did not vary on family history risk or genetic risk levels (45).

The perception of the magnitude of risk can also be observed through the worry caused by type 2 diabetes risk. Those with low risk were significantly less worried than participants who had a higher risk for type 2 diabetes, but there was no statistical significance of the type of risk given (traditional risk information or genome-wide polygenic risk score) (44). In general, worry was eased by the knowledge that type 2 diabetes was a manageable and ‘not as life-threatening’ disease (24).

#### 3.3.1 Increased risk perception

A family history of type 2 diabetes was associated with an increased perceived risk of developing type 2 diabetes (21, 28, 32, 42, 45–47), but this had no effect on the perceived seriousness of the disease (48). Genetic risk though did not correlate with the perception of developing diabetes during the lifetime. However, those who had more genetic risk alleles were more likely to perceive a more serious risk for type 2 diabetes. This change was strongest with those who had an average and moderate family history, but those with a high family history risk had no statistically significant changes in risk perception (45). Those who emphasized genetic risk as a causal explanation assessed their risk higher than those who emphasized behavioral risk as stronger. Despite the high-risk perception, they perceived type 2 diabetes as a preventable disease. Those who were not clear about the genetic factors assessed their risk at the lowest level, and as well as being less concerned about developing type 2 diabetes, saw it as a manageable disease (33).

#### 3.3.2 The accuracy of the risk perception

There were two kinds of trends relating to the accuracy of the risk perception (Table 3). Firstly, most participants underestimated their overall risk compared to a clinical overall risk assessment for diabetes. Although several participants concordantly or overestimated their individual risk factors, they still underestimated their overall risk (24). Additionally, a separate study highlighted a tendency towards a more consistent or underestimation of risk perceptions rather than overestimation (43). Conversely, people tended to overestimate rather than underestimate their risk in one study. Also, the risk assessment became more accurate immediately after the risk assessment result, but the accuracy weakened again at 8 weeks. Those who overestimated their type 2 diabetes risk continued to overestimate the risk after receiving a risk estimate, although more accurately than was seen at baseline. Those who underestimated their type 2 diabetes risk at baseline perceived their risk more accurately after receiving a risk estimate, and continued to perceive their risk more accurately at 8 weeks post-intervention. However, those who were actually at higher risk and received a higher risk estimate were more likely to underestimate their risk. These participants also received a genotypic or phenotypic risk estimate for type 2 diabetes, but there were no differences in risk perception between these risk information types (49).

**Table 3:** Personal risk perception compared to actual risk assessment.

Ref. no., Authors, Year	*n*	Personal risk perception	Actual risk assessment	Underestimated risk perception	Concordant/ accurate risk perception	Overestimated risk perception
49 Silarova et al. (2018)	379	How likely are you to get type 2 diabetes in your lifetime? Scale 0–100	Cambridge Diabetes Risk score	24.1%	1.3%	74.5%
24 Daack-Hirsch et al. (2020)	153	PRF-T2DM and perceived overall risk	Clinical overall risk assessment for diabetes	73%	26%	1%
43 de la Haye et al. (2021)	49	Increased risk/average risk	Families SHARE algorithm	42.5%	47.5%	5.0%

The relationship between actual risk and perceived risk was described in a few other studies as well. Those participants who were at low actual risk perceived their risk to be lower than those with higher risk levels, with no significant differences based on the type of risk information provided (traditional risk factors or additionally genome-wide polygenic risk) (44). In addition, those who had an elevated or high actual diabetes risk and perceived themselves at increased (i.e., moderate or high) diabetes risk were found to have a significant association with a family history of diabetes (46).

## 4 Discussion

### 4.1 Summary of main findings

Hereditary risk was well recognized as a risk factor for type 2 diabetes. Although, of course, the review included studies that dealt with hereditary risk, it was quite often mentioned as the most known risk factor. Although heredity was strongly seen as a risk factor for type 2 diabetes, the disease was perceived as preventable and manageable.

Type 2 diabetes became salient when a family member had diabetes. In this case, the perception was based on experience, and the desire to do something different arose by comparing one’s own health behavior to the behaviors of others. In contrast, the perception of family history and genetic/genomic risk increased awareness of health behavior and the intention to make changes, yet actual practical changes remained infrequent. Something similar has emerged previously as well. Genetic risk information did not increase motivation to change lifestyle to prevent diabetes (50). Furthermore, participants who predominantly perceived type 2 diabetes to genetic causes believed that prevention was beyond their control (5).

This systematic review included only a small degree of coverage about citizens’ perceptions of genetic/genomic risk, especially when considering how much direct-to-consumer genomic testing has increased. However, genetic/genomic risk should be discussed in healthcare more broadly than just genetic risk or a polygenic risk score, since genetic risk is contextualized more in familial and social narratives than in mathematical models (15). This also confirms the view expressed in this review, that personalized pedigree and diseased risk in the community were more salient than information about the level of risk (43). So, both genetic scores and any risk levels are better understood within the context of family experience, rather than as mere numerical values or levels.

Ethnic risk is one of the inherited risks that arose, but there is very little research on the perception of ethnic risk. The study of how the diabetes epidemic has been constructed in the UK media in 1993, 2001 and 2013 shows that there has been a transition from medical to behavioral and then to societal as a cause of type 2 diabetes. Especially in 2013, race, ethnicity and culture were stated as ‘high risk’ factors for diabetes in the UK media. Some ethnic groups were considered to have a higher risk of type 2 diabetes than others. Also, considering diabetes as an “epidemic” could shift the responsibility from the individual to society (13). If ethnic risk is emphasized in the media, the aim of public health promotion may turn oppositely into a strengthening of the perception of the inevitability of disease, and increase the perception that even healthy lifestyles cannot prevent disease. However, understanding the factor of ethnic risk can enable the targeting of public health programs aimed at preventing type 2 diabetes.

Perceptions of inherited cultural and health behavior show that hereditary risk has been understood more widely among citizens than is usually thought in healthcare. Pijl et al. also mentioned an inherited lifestyle, with diet in particular being perceived as a hereditary factor (5). If the citizen sees health behavior risks as heritable, risk management and preventive actions are also seen in a different light than feasible changes in health habits. Once more, family unit seems to play a significant role in the formulation of the perception of risk, but also in how the existing risk can be managed.

The perception of the magnitude of personal risk varied because of causal explanation, personal risk level, and family history. A Family history was strongly associated with increased perceived risk. Different trends were obtained regarding the accuracy of risk perception and actual risk, although, the results were not comparable because risk perception was measured in different ways in these studies. However, there has also been a lack of congruency between perceived and actual diabetes risk in the general population (51).

Although the themes were not formed according to the risk perception concept analysis (9), these dimensions were visible in the systematic review. For example, the concept of personal risk formed while recognizing one’s own family history as a risk factor, and perceived severity through family experiences while witnessing significant events, complications, and diabetes management. Perceived likelihood could be assessed by comparing one’s health habits and other risk factors with those of family members who had type 2 diabetes, and an optimistic bias came up while underestimating the level of personal risk. Finally, the affective dimension arose, for example, when type 2 diabetes caused worry about the wellbeing of offspring. The visibility of these dimensions confirmed that the risk perception approach used in this systematic review covered the areas that have been previously studied about risk perception.

### 4.2 Limitations

There are limitations, but also strengths in this systematic review. The review was conducted systematically using the PRISMA guidelines and by accessing reliable databases. Record selection and quality assessment were undertaken independently by two authors, and differing opinions were discussed until a consensus was reached. Coding, analysis, and the compilation of the manuscript were performed by one author, but all the authors contributed valuable comments and suggestions for corrections.

The systematic review reveals a limited amount of research on specific topics, notably the perception of genomic and ethnic risk. Given that the utilization of genomic risk information is relatively new and continually evolving, it is crucial to continually update our understanding of how citizens perceive genomic risks. Furthermore, measuring risk perception is multifaceted, emphasizing the need for consistent methods and scales when examining the disparity between perceived and actual risks. Without such standardization, the results of studies may lack comparability, as demonstrated in this review.

One of the conditions related to the screening was that type 2 diabetes could be interpreted separately in the overall results of the study (Table 1). In a study by Rego et al. (40), the genome test also covered other multifactorial diseases, but only type 2 diabetes was taken into account in this review. Furthermore, Cunningham et al. (30) did not differentiate between type 1 and type 2 diabetes in their study, but type 2 diabetes was understood and interpreted through authentic quotes. As such, the screening required interpretation, and bias may also have occurred in its reporting.

In thematic analysis, the researcher has a role in the interpretation of the data. In this review, some sections had to be clarified so that the results can be better understood when compared to other studies. The themes could still be interpreted more deeply, but the results also give room for their interpretation in different healthcare contexts.

## 5 Conclusion

The systematic review provides a new perspective on the perceptions of the inherited risk for type 2 diabetes, which can enhance the comprehension of risk perception in general within healthcare. Effective risk communication is an important part of preventive health care. Inherent to this, it is critical to identify those who have intentions to modify their health behaviors, as well as those who perceive themselves as powerless in avoiding the disease. It is also imperative to clarify the origins of such thinking patterns, thereby enabling the implementation of targeted and efficient risk communication and prevention strategies to address these underlying causes.

One recommendation is to incorporate more family-centered interventions into current personalized healthcare practices. Encouraging the involvement of the entire family could bolster public health outcomes, and fostering open dialog could further promote the view of type 2 diabetes within the context of the entire family. This approach encourages a collective family-based approach to disease management.

## Data availability statement

The original contributions presented in the study are included in the article/supplementary material, further inquiries can be directed to the corresponding author/s.

## Author contributions

EA: Conceptualization, Data curation, Formal analysis, Methodology, Validation, Visualization, Writing – original draft. ML: Data curation, Writing – review & editing. AH: Supervision, Writing – review & editing. MK: Supervision, Writing – review & editing.
